# Unlocking the Value of Birch Bark: Betulin as a Sustainable Additive for Advancing Cellulosic Material Performance

**DOI:** 10.1021/acssusresmgt.5c00562

**Published:** 2025-11-20

**Authors:** Armando Córdova

**Affiliations:** † FSCN Research Center, Organic Chemistry, Mid Sweden University, Holmgatan 10, 851 70 Sundsvall, Sweden

**Keywords:** betulin, cellulosic materials, wet strength, hydrophobic, eco-friendly, synergy, sustainable additive

## Abstract

This Viewpoint explores betulin, a naturally occurring medicinal pentacyclic triterpene extracted from birch bark, a forestry side stream, as a sustainable additive for improving the performance of cellulosic products.

## Toward Holistic Resource Management

As the world transitions toward a circular economy and sustainable materials management, renewable resources such as cellulose are gaining traction as substitutes for fossil-based materials.^1–3^ However, the performance limitations of cellulose-based products often necessitate the incorporation of functional additives, many of which are still derived from non-renewable sources and are toxic and harmful.^4,5^ This reliance hinders the overall sustainability of such products. There is a critical need for natural, biodegradable, and effective additives that can enhance material properties without compromising environmental compatibility.

Betulin, a medicinal pentacyclic triterpene is primarily found in the outer bark of birch trees (genus *Betula*), which serves as its main natural source and represents a compelling candidate for sustainable applications (Figure 1).^6,7^ It is also present in birch sap and, in smaller amounts, in other plants such as hazel bark, calendula, and certain species of thistle. As a byproduct of the forestry industry, betulin is largely underutilized, typically discarded during timber processing. Its unique chemical structure imparts properties such as hydrophobicity and antimicrobial activity, making it particularly attractive for integration into cellulosic systems (Figure 1).^8–10^ This Viewpoint explores the potential of betulin as a sustainable, multifunctional additive for enhancing cellulose-based materials.

**Figure 1:**
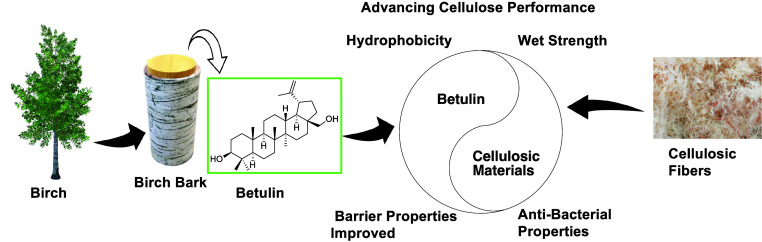
Schematic overview illustrating the process from birch tree to birch bark, where betulin is located and extracted, followed by its integration with cellulosic materials to enhance their performance.

## Betulin: A Forestry Side Stream with Untapped Potential

The birch bark, often treated as low-value waste or burned for energy, contains betulin as a major component, comprising up to 30% of the dry weight of the outer bark.^6,7b^ Betulin has several important properties such as biological activity (e.g. antitumor, antimicrobial, antiviral, hypolipedemic),^7a,8,11–16^ hydrophobicity^8–10,17–20^ and can undergo interesting morphological changes.^9,21–23^ The valorization of this forestry side stream aligns with principles of sustainable resource management, including biomass cascading and circular utilization of natural resources.^24,25^ By extracting betulin from birch bark using green methods (e.g., supercritical CO_2_, ethanol-based solvents), it is possible to generate high-value products while reducing industrial waste.^24^ This approach fits within broader bioeconomy strategies aimed at maximizing the value derived from forest biomass. Importantly, sourcing betulin does not compete with food systems or require dedicated land use, further enhancing its sustainability profile.

## Enhancing Cellulosic Product Performance Sustainably

Cellulose-based materials, such as paper, molded fibers, and nonwovens, often face challenges related to moisture sensitivity, microbial degradation, and barrier limitations. Betulin can address these weaknesses through its natural functionality:

- *Hydrophobicity:* The lipophilic nature of betulin makes it an excellent moisture barrier. When applied as a surface treatment or coating, it reduces water absorption and improves dimensional stability.
- *Antimicrobial properties:* Betulin has demonstrated bioactivity against a range of microbes, offering potential for hygienic packaging, personal care products, and food-contact applications.
- *Barrier enhancement:* Betulin-containing coatings can improve resistance to water vapor, oils, and other environmental stressors, enhancing the durability of cellulosic packaging.

These benefits are particularly relevant in sectors aiming to replace plastic with renewable alternatives, making betulin a strategic tool in sustainable packaging and product development. It is noteworthy that it was recently discovered that betulin can be used as a strength agent significantly improving the wet strength of cellulosic materials when synergistically combined with hot pressing.^26^ In this system, the colloidal betulin particles in the aqueous suspension underwent polymorphic transformation from prismatic crystals to orthorhombic whiskers, which subsequently intertwined with the cellulosic fibers.

## Circular Design and End-of-Life Benefits

A critical consideration in sustainable materials management is the behavior of additives at the end of a product’s life. Betulin, being a naturally occurring molecule, does not impede the compostability or biodegradability of cellulose.^27^ Betulin, as a naturally occurring pentacyclic triterpenoid, is considered biodegradable under environmental conditions, particularly in soil or compost systems where microbial activity is present. Its degradation primarily proceeds via microbial enzymatic oxidation, initiated at the hydroxyl groups at C-3 and the isopropenyl side chain. This process leads to the formation of intermediate products such as betulinic acid**,** betulonic acid, and ultimately to smaller aliphatic acids. This contrasts with many synthetic additives, which can complicate recycling or lead to microplastic pollution. In composting environments, betulin can degrade alongside cellulose, enabling truly circular product design. Its use supports frameworks such as “design for environment” (DfE) and “design for disassembly,″ facilitating easier reintegration of materials into natural cycles.

## Pathways for Industrial Integration

At present, betulin is employed in industry either in its crystalline form or as birch bark extracts rich in betulin, primarily for pharmaceutical and cosmetic applications. From a processing standpoint, betulin is compatible with various methods of application, including solvent-based coatings, wax emulsions, and direct blending into pulp slurries. Its versatility allows it to be introduced during multiple stages of product fabrication, whether in papermaking, fiber molding, or film coating. Challenges remain in standardizing extraction techniques, ensuring consistent purity, and optimizing formulations for industrial use. A major cost factor in the extraction of betulin is the recycling of ethanol, which is used as the primary solvent. This, together with the overall efficiency of the extraction process, significantly affects the economic viability of large-scale production.^27^ However, with increasing interest in green chemistry and low-impact processing, the path forward for scalable betulin integration is promising. In this context, the recent use of colloidal betulin particles for cellulose modification in water-based systems opens for green industrial applications.^26^ Another way is the use of solvent free organocatalytic methods of cellulose modification,^28,29^ which have utilized Betulin as a modifying agent.^8^

## Challenges and Research Opportunities

Despite its potential, several areas require further exploration:

- *Life Cycle Assessment (LCA):* Comprehensive environmental assessments are needed to quantify the true benefits of using betulin versus synthetic additives.
- *Extraction efficiency:* Research into low-energy, high-yield extraction methods can improve the economic feasibility of betulin utilization.
- *Regulatory approval:* Especially for food packaging or personal care products, safety and toxicology studies may be required.
- *Material synergy:* Combining betulin with other bio-derived materials (e.g., chitosan, lignin) could create multifunctional, high-performance composites.

## A Step toward Resource-Wise Innovation

Betulin exemplifies how overlooked natural compounds can offer elegant solutions to modern sustainability challenges. Its integration into cellulosic products enhances performance while reinforcing principles of resource efficiency, waste minimization, and circularity. As industries seek greener alternatives that do not sacrifice functionality, betulin represents a path forward that bridges forest resource valorization with next-generation material design. Encouraging its development and adoption could significantly advance the sustainable management of both natural resources and the materials derived from them. This Viewpoint aims to catalyze interdisciplinary collaboration around betulin-based innovations and invites further exploration into its role in sustainable resource management.
